# 

**Published:** 1883-12

**Authors:** 


### CONTENTS:

Art. I.—Dental Nomenclature........................337

Art. II.—Dr. Genese's Articulator........................344

Art. III.—Steamer for Dental Purposes........................348

Art. IV.—Some Points in Oral Surgery of Interest to the General Practitioner. By George L. Parmele, M. D., D. D. S. (Continued)........................349

Art. V.—Mechanical Treatment of Cleft Palate, Obturators, and Artificial Velum. By Oscar Booth, D. D. S........................357

Art. VI.—Pericementitis. By George A. Mills, D. D. S. (Continued)........................363

Art. VII.—Overcoming Heredity. By Mrs. M. W. J........................368

Art. VIII.—Anomalies in the Eruption of the Teeth of Man. By Dr. Magitot........................372

Art. IX.—Case of Antral Abscess of Twenty Years Standing. By Thomas Bryant, F. R. C. S........................375

Art. X.—Practical Knowledge........................377

Dental Department of the University of California........................379

Maryland and District of Columbia Dental Association........................380

### EDITORIAL, ETC.

Dental Students in Baltimore City........................380

Maryland State Dental Association........................381

The Proposed Bill to Regulate the Practice of Dentistry in the State of Maryland...381

Visiting Lists for 1884—

Physician's Visiting List........................382

Physician's Daily Pocket Record........................383

Medical Record, Visiting List, and Physician's Diary........................383

A New Work on Dental Medicine........................383

